# RALYL Overexpression Suppresses Colorectal Cancer via Modulating HNRNPC‐Mediated MNK2 Alternative Splicing

**DOI:** 10.1002/cnr2.70179

**Published:** 2025-03-07

**Authors:** Zenghui Ma, Jianbin Zhu, Min Chen, Guangming Wu, Xiaolong Liu, Zhanjun Hu, Yufei Feng, Xiaoliang Wang, Feng Liu

**Affiliations:** ^1^ Department of Pediatric Surgery Children's Hospital of Fudan University, National Children's Medical Center Shanghai China; ^2^ Department of Hepatobiliary Surgery, Pudong Hospital Fudan University Shanghai China; ^3^ Department of General Surgery The People's Hospital of Wuhai Inner Mongolia Wuhai Inner Mongolia China

**Keywords:** alternative splicing, colorectal cancer, HNRNPC, MNK2, RALYL

## Abstract

**Background:**

Colorectal cancer (CRC) stands as the second most prevalent cause of cancer‐related mortality globally, while its incidence holds the third position among newly diagnosed cancer cases worldwide. Colorectal carcinogenesis is complicated, and the processes are triggered by the complex interaction of some genetic and environmental factors, including DNA methylation. Previous studies showed that RALYL is hypermethylated in CRC. We aimed to explore the role of RALYL in CRC involved in MNK2 alternative splicing in the present study.

**Methods:**

Bioinformatics analysis, detection in CRC samples, and experiments in vitro and in vivo combined with gene knockdown and overexpression were conducted. Cell proliferation and tumor growth assays were performed.

**Results:**

Results showed that hypermethylated RALYL is lowly expressed in CRC. Overexpression of RALYL suppresses cell proliferation in vitro and tumor growth in vivo in CRC. MNK2 alternative splicing is essential for the tumor suppressive role of RALYL, along with RALYL regulating MNK2 alternative splicing via HNRNPC in CRC.

**Conclusions:**

RALYL potentially exerts an inhibitory effect on CRC by engaging with HNRNPC to orchestrate the alternative splicing of MNK2. RALYL binds to HNRNPC to promote MNK2 splicing into MNK2a instead of MNK2b, consequently activating the p38 MAPK signaling pathway and inhibiting tumor proliferation in CRC. Our findings suggest that RALYL might suppress CRC through binding to HNRNPC to promote MNK2 splicing toward MNK2a, thereby activating the p38 MAPK signaling cascade.

**Abbreviations**
CCK8: Cell Counting Kit‐8
COAD: colon adenocarcinoma
CRC: colorectal cancer
IHC: immunohistochemistry
READ: rectum adenocarcinoma
SD: standard deviations

## 1 Introduction

Colorectal cancer (CRC) is the second leading cause of cancer‐related death, and its morbidity ranks third in terms of newly diagnosed cancer cases globally [1]. The etiology of colorectal carcinogenesis is intricate, stemming from the complicated interplay of various genetic and environmental factors, including sedentary behaviors, DNA methylation patterns, and the gut microbiota [1, 2, 3]. Therefore, comprehension of the pathophysiological mechanisms of oncogenesis progress may serve as a novel therapeutic target of CRC.

RALYL is one of the hypermethylated genes obtained from methylated DNA immunoprecipitation (MeDIP‐seq) to detect the methylation profile of cell‐free DNA (cfDNA) in patients with CRC in our previous study [4]. RALYL suppressed ovarian cancer progression by regulating MAPK/CDH1 signaling pathways [5]. Analysis of the UALCAN database further revealed that RALYL undergoes hypermethylation in both colon adenocarcinoma (COAD) and rectum adenocarcinoma (READ) [6], accompanied by a downregulation of its expression in these cancer types [6, 7].

The kinases MNK1 and MNK2 play an important role in cancer, and targeting them has emerged as a valuable strategy in oncology [8]. *MKNK2* encoding for MNK2 is alternatively spliced to yield two splicing isoforms of MNK2a and MNK2b. It is MNK2a instead of MNK2b that contains a MAPK‐binding domain to suppress colon cancer by regulating the p38 MAPK pathway [9]. According to another study, regulating the alternative splicing of *MKNK2* by binding to MNK2 pre‐mRNA at e14a induces down expression of MNK2a splicing and regulates the p38 MAPK/PPARα signaling pathway to promote gastric cancer peritoneal metastasis [10]. It was further indicated that the tumor suppressive activity of MNK2a is due to its colocalization with p38a‐MAPK in the nucleus and thus, the alternative splicing of *MKNK2* to downregulated MNK2a, which is a tumor suppressor mechanism and is lost in some types of cancers such as breast, lung, and colon carcinoma [9].

We aimed to explore the role of RALYL in CRC involved in MNK2 alternative splicing in the present study. Bioinformatics analysis, detection in CRC samples, and experiments in vitro and in vivo combined with gene knockdown and overexpression were conducted. MNK2 associated with its alternative splicing is proposed to serve as a potential target in CRC therapy.

## 2 Materials and Methods

### 2.1 Reagents and Antibodies

Adezmapimod (SB203580) (HY‐10256) was purchased from MedChemExpress (Shanghai, China). Adezmapimod is a specific p38 MAPK inhibitor that can permeate cells, inhibit p38 MAPK, and inhibit the subsequent activation of MAPKAP kin‐2 and MAPKAP kin‐3. By inhibiting p38 MAPK, SB203580 can effectively inhibit part of the signal transduction induced by some inflammatory factors (such as IL‐1β, TNF‐α). Crystal violet (C0121), Trizol (R0016), ECL chemiluminescence reagent (P0018S), RIPA buffer (P0013C), BCA protein detection kit (P0010), Lipo6000 (C0526), Cell Counting Kit‐8 (CCK8, C0037), and BeyoClick EdU‐555 kit (C0075S) were supplied by Beyotime (Shanghai, China). Primary antibodies of HNRNPC (11760‐1‐AP), RALYL (17179‐1‐AP, 67534‐1‐Ig), GAPDH (60004‐1‐Ig), and applicable secondary antibodies were purchased from Proteintech (Wuhai, China). RALYL overexpression and negative control lentivirus or vectors were supplied by Shanghai Yuanke Biotechnology Co. Ltd. siRNAs and oligonucleotides were purchased from Sangon Biotech (Shanghai) Co. Ltd.

### 2.2 Patients and Tissue Samples

Thirteen pairs of tumors and adjacent normal tissues were collected from July 2021 to December 2022 in The People's Hospital of Wuhai Inner Mongolia following the hospital's Ethics Committee, and all the patients enrolled in the present study signed informed consent. The patients were anonymously coded, complying with ethical guidelines, as instructed by the Declaration of Helsinki.

### 2.3 Cell Culture and Transfection

CRC cell lines of SW480, HCT116, SW620, and DLD1 were supplied by Cell Bank, Chinese Academy of Sciences. Human normal colonic epithelial cells NCM460 were purchased from the American Type Culture Collection (Manassas, Virginia, USA). SW480 and SW620 cells were cultured in L‐15 (11415064, Gibco) medium containing 1% penicillin/streptomycin and 10% fetal bovine serum (Gibco, USA) at 37°C in an incubator. HCT116 cells were cultured in McCoy's 5a medium (12330031, Gibco) supplemented with 1% penicillin/streptomycin and 10% fetal bovine serum at 37°C in a 5% CO_2_ incubator. DLD1 cells were cultured in RPMI 1640 medium (11875093, Gibco) supplemented with 1% penicillin/streptomycin and 10% fetal bovine serum at 37°C in a 5% CO_2_ incubator. NCM460 cells were cultured in Dulbecco's modified Eagle's medium (KeyGEN BioTECH, Jiangsu, China) with 10% fetal bovine serum (Gibco, USA) at 37°C in a 5% CO_2_ incubator.

For experimental purposes, cells were transfected with overexpression vectors to upregulate target gene expression, siRNA to knockdown specific gene expression, block oligonucleotides to inhibit mRNA translation, and a negative control to ensure the specificity of the observed effects. All transfections were performed using the Lipo6000 transfection reagent, following the manufacturer's protocols for transient transfection.

### 2.4 In Vivo Mouse Xenografts and Treatment

All the animal experiments in the present study were conducted as per the Experimental Animal Ethics Committee of The People's Hospital of Wuhai Inner Mongolia. The mice xenograft model was made according to the previous study [11] with minor modifications. SPF grade, 6–8 weeks old, male Balb/c nude mice were used in our study and kept under a sterile environment at 25°C ± 2°C, with 50%–60% humidity and a 12 h light/dark cycle. All the animals were free to water and food. Following a week of adaptive feeding, each mouse underwent a subcutaneous injection of 5 × 10^6^ human CRC cells suspended in 200 μL of PBS, administered into the underarm region of the right forelimb. One week after the inoculation, the tumor‐bearing animals were randomly divided into two groups with five mice in each group according to treatment with vector or RALYL overexpression lentivirus with tail intravenous injection. The survival of the animals was observed, and the tumor size was measured every 3 days.

### 2.5 Western Blotting

Total protein was extracted from tissue or cells using RIPA buffer supplemented with protease inhibitors to prevent protein degradation. The lysates were then subjected to centrifugation to collect the protein supernatant, which was quantified using a BCA protein detection kit to ensure accurate protein concentration measurement. For electrophoresis, 20 μg of protein from each sample was separated on 6%–15% polyacrylamide gels, allowing for the resolution of proteins of varying sizes. Following electrophoresis, the proteins were transferred onto PVDF blotting membranes (ISEQ00010, Millipore, USA) for subsequent immunodetection.

The membranes were blocked to prevent nonspecific binding and then incubated overnight with primary antibodies specific for HNRNPC (1:1000), RALYL (1:1000), and GAPDH (1:5000) to ensure the detection of target proteins. After washing, the membranes were incubated with HRP‐conjugated secondary antibodies, which bind to the primary antibodies. The presence of the target proteins was detected using an ECL chemiluminescence reagent, and the signals were visualized and captured using a chemiluminescence imager. This method ensured the specific and sensitive detection of HNRNPC, RALYL, and GAPDH in the protein samples.

### 2.6 RT‐qPCR

The total RNA of cells was extracted using Trizol reagent according to the users' instructions. The RNA was subsequently reverse‐transcribed into cDNA. RT‐qPCR experiments were conducted based on the cDNA on the instrument of real‐time PCR (ABI7500). GAPDH is used as an internal control. The relative expression of the target genes was analyzed using the 2^−∆∆Ct^ method. The primer sequences used in the present study were as follows: RALYL forward: GCGCCTGGAGAAGATTGAGA, reverse: GAAACAGCTCATGACCCCCA; MNK2a forward: TCCGTGACGCCAAGCAG; reverse: GGTCTTTGGCACAGCTG; MNK2b forward: TCCGTGACGCCAAGCAG, reverse: GAGGAAGTGACTGTCCCAC; GAPDH forward: GGAGCGAGATCCCTCCAAAAT, reverse: GGCTGTTGTCATACTTCTCATGG.

### 2.7 CCK8

Cell viability was assayed using CCK8 according to the manufacturer's instructions. Briefly, 1 × 10^4^ cells/well were plated into a 96‐well plate and allowed to grow for 12 h. After transfection with overexpression/siRNA/negative vector, MNK2a block 2′‐OMe oligos, the cells were then continued to be cultured for 24 h. Ten microliter CCK8 reagent was added to the cells at the end time and incubated at 37°C for 4 h, and the absorbance value was detected at 490 nm by a microplate reader.

### 2.8 Colony Formation

Cell proliferation was assessed using a colony formation assay. Specifically, 1 × 10^3^ cells were plated in 2 mL of medium per well in six‐well plates and allowed to adhere for 12 h. Following this incubation period, the cells were transfected with either overexpression vectors, siRNA, or a negative control vector. After a 6‐h transfection period, the medium was replaced with fresh medium to remove transfection reagents and ensure optimal cell growth conditions.

The medium was subsequently changed every 3 days to maintain nutrient availability and optimal growth conditions for the cells. The cells were cultured for a total of 14 days to allow for colony formation. At the end of the experiment, the medium was carefully removed, and the cells were gently washed to remove any residual medium or dead cells. The colonies were then stained with crystal violet for observation.

Following staining, the cells underwent rinsing with distilled water to eliminate any excess dye. Subsequently, images of the stained colonies were acquired and subjected to analysis utilizing ImageJ software, enabling the quantification of both colony numbers and sizes. This approach served as a metric to assess cell proliferation and the efficacy of the transfection processes.

### 2.9 EdU

EdU staining was conducted to detect cell proliferation according to the manufacturer's instructions. Initially, cells were plated on a coverslip and allowed to grow into a single‐cell layer for 48 h. Following this incubation, EdU was added to the cell culture and incubated at 37°C for 2 h to allow incorporation of EdU into the newly synthesized DNA of proliferating cells.

After the incubation period, the cells were fixed using 4% paraformaldehyde to preserve cell morphology and immobilize the cellular components. Click Additive Solution was then added to the cells to facilitate the reaction between the EdU and the fluorescent azide, allowing for the visualization of EdU incorporation.

Subsequently, the cells were stained with DAPI to label the nuclei, providing a contrast for identifying cell nuclei against the EdU staining. Images of the stained cells were captured using a fluorescence microscope, enabling the visualization and analysis of cell proliferation based on EdU incorporation.

### 2.10 Immunofluorescence

The cells were initially plated on a coverslip and allowed to grow into a single‐cell layer. After reaching the desired confluence, the cells were fixed with 4% paraformaldehyde for 30 min to preserve cell structure. Following fixation, the coverslip was washed with PBS to remove any residual fixative.

The cells were then incubated with primary antibodies against HNRNPC and RALYL to detect the presence and localization of these proteins. This was followed by incubation with fluorescently labeled secondary antibodies, which bind to the primary antibodies and allow for the visualization of the target proteins under a fluorescence microscope.

DAPI was used to stain the cell nuclei, providing a clear contrast for identifying cell structures. Fluorescent images of the cells were then captured using a fluorescence microscope, allowing for the analysis of HNRNPC and RALYL expression and localization within the cells.

### 2.11 Immunohistochemistry (IHC)

The protein of Ki67 and RALYL was detected utilizing immunohistochemistry staining based on the previous study [12]. Initially, human CRC‐adjacent normal tissue and mouse tumor tissue were fixed in 4% paraformaldehyde and then embedded in paraffin blocks. Paraffin sections were made and followed by dewaxing, antigen repair, blocking endogenous peroxidase, serum blocking, primary antibody incubation, secondary antibody incubation, DAB color development, Harris hematoxylin restaining of the nucleus, and film sealing in turn. Microscopic examination was then performed, and images were collected and analyzed.

### 2.12 Bioinformatics Analysis

RALYL promoter methylation levels in COAD and READ were obtained from the UALCAN database (https://ualcan.path.uab.edu/); RALYL expression in COAD and READ was analyzed from GEPIA (http://gepia.cancer‐pku.cn/) and TCGA. The interaction between RALYL and HNRNPC is presented in The Interaction section of The Human Protein Atlas database. The interacting protein HNRNPC of RALYL was predicted by the String and GPS‐Prot databases.

### 2.13 Statistical Analysis

All the data were analyzed using Graph Pad Prism 9 and ImageJ software. The data were expressed by means ± standard deviations (SD) from at least three independent experiments. The difference was analyzed using Student's *t*‐test or ANOVA. When the *p* value was less than 0.05, the difference was considered significant.

## 3 Results

### 3.1 Hypermethylated RALYL Is Low Expressed in CRC

**FIGURE 1:**
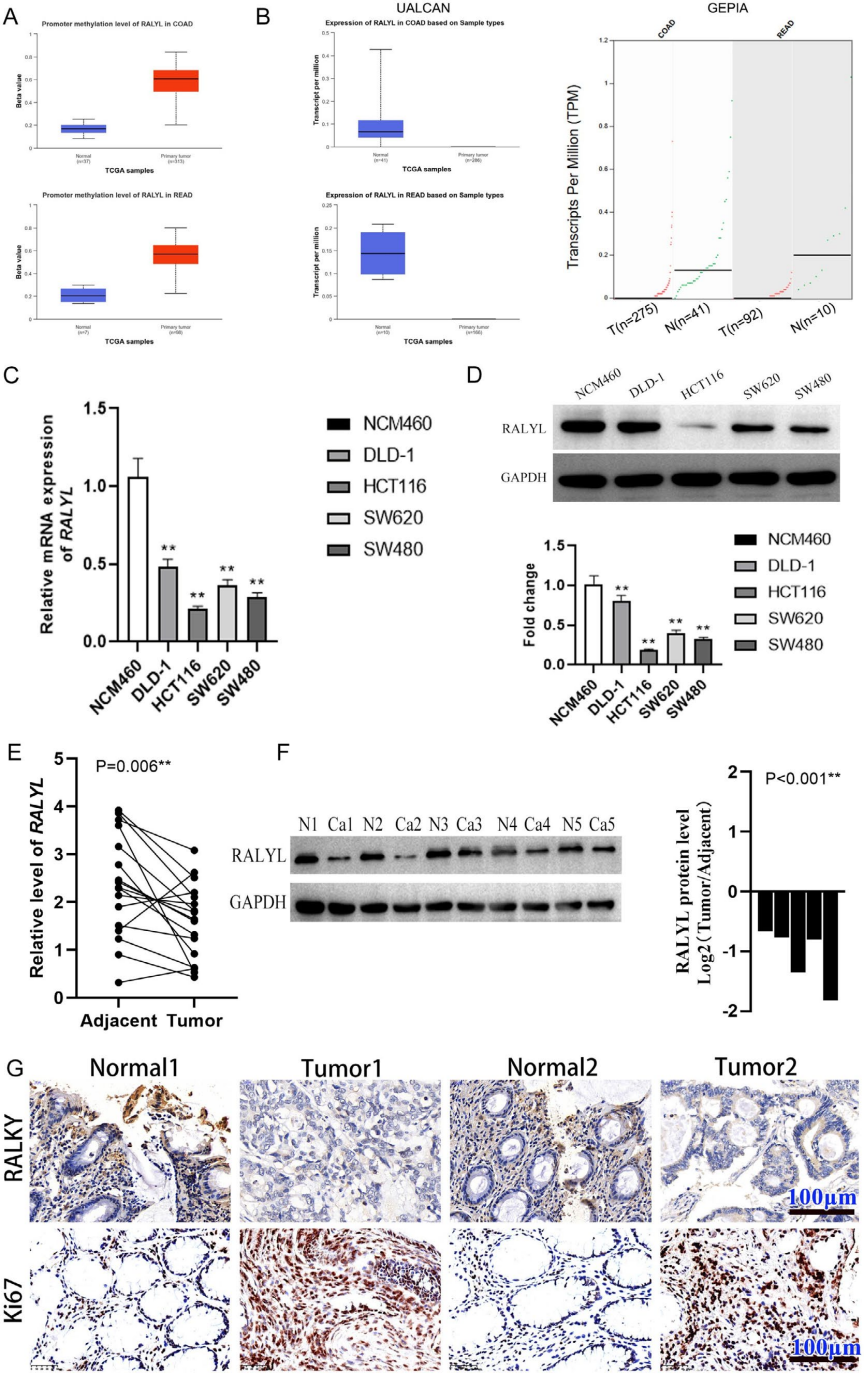
The methylation and expression of RALYL in CRC. (A) RALYL promoter methylation levels in COAD and READ in UALCAN database. (B) The RALYL level in COAD and READ in the UALCAN and GEPIA database. (C and D) Relative mRNA and protein level of RALYL in human normal colonic epithelial cell NCM460 and colorectal cancer cell lines (SW480, HCT116, SW620, DLD10) (*n* = 3). (E) Relative expression of RALYL in CRC tumor and the adjacent tissues using RT‐qPCR (*n* = 17). (F) Protein level of RALYL in five cases of CRC using western blotting. (G) Representative micrographs of HE and IHC of RALYL and Ki67 in colorectal cancer. **p < 0.01. COAD, colon adenocarcinoma; CRC, colorectal cancer; HE, hematoxylin and eosin; IHC, immunohistochemistry; READ, rectum adenocarcinoma.

Results of bioinformatics analysis showed that RALYL was hypermethylated in COAD and READ (Figure 1A). Analysis of data from TCGA using UALCAN and GEPIA showed that RALYL expression was low in primary tumor in COAD and READ compared with normal tissue (Figure 1B). The expression of RALYL was then assayed in CRC cell lines along with normal colonic epithelial cells, the results of which indicated that low mRNA and protein expression of RALYL was observed in all the CRC cells compared with normal colonic epithelial cells (Figure 1C,D). Results of human CRC samples assay for mRNA and protein demonstrated that RALYL was downregulated in tumor tissue compared with adjacent normal tissue (Figure 1E,F). It was confirmed by IHC assay that RALYL expression was lower in the tumor than in adjacent normal tissue, which was negative with the expression of Ki67, a cell proliferation‐related marker (Figure 1G).

### 3.2 Overexpression of RALYL Suppresses Cell Proliferation In Vitro and Tumor Growth of Colorectal In Vivo in CRC

**FIGURE 2:**
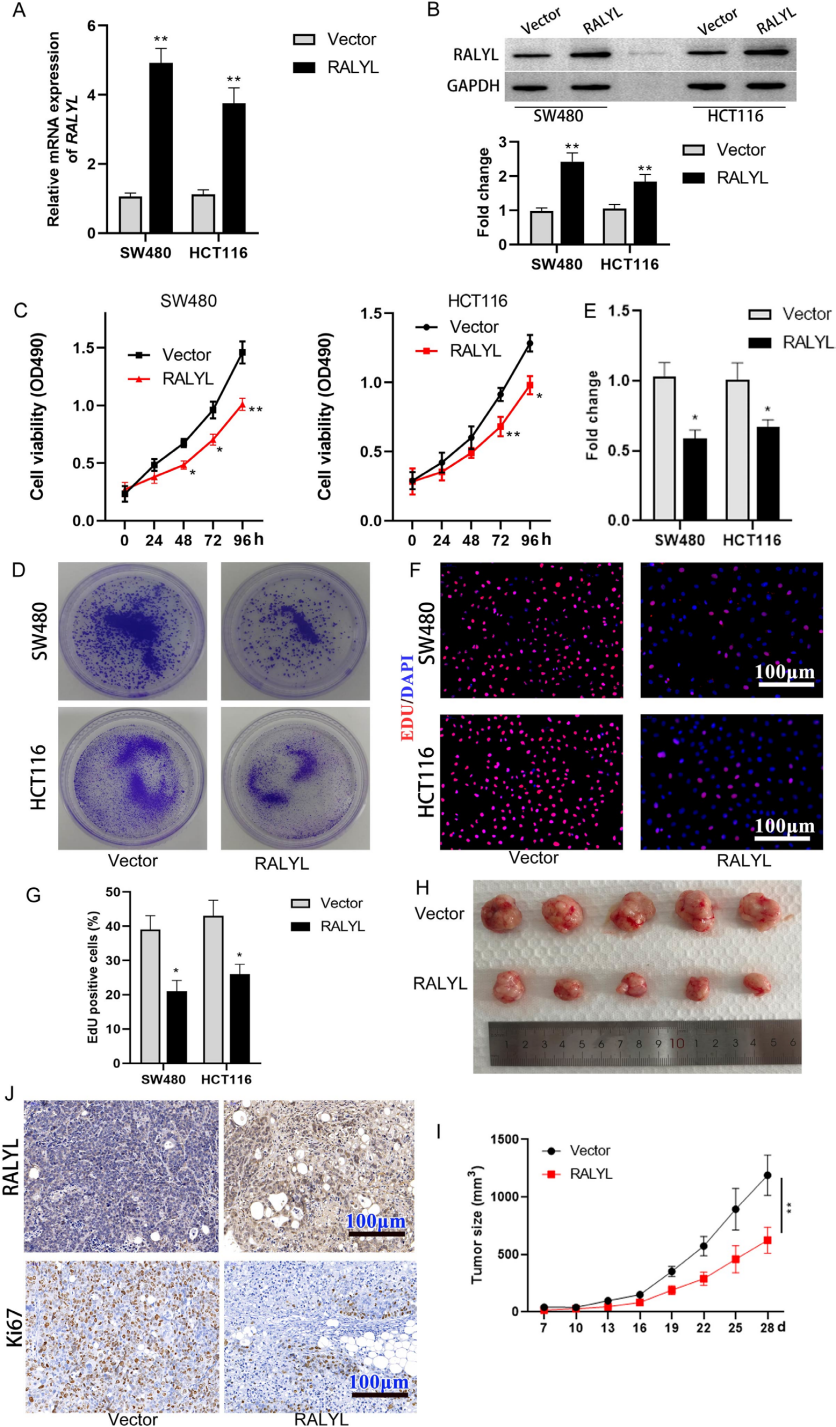
Effect of RYLYL overexpression on CRC cell growth. (A and B) The mRNA and protein levels of RALYL in cells transfected with pcDNA3.1‐RALYL (RALYL group) or the vector control group. (C) MTT assays are used to determine cell proliferation in transfected SW480 and HCT116 cells. (D) Colony formation assay evaluates the in vitro colony formation of transfected cells. (E) Percentage of EdU‐positive cells in CRC cells. *n* = 3. *p < 0.05, **p < 0.01 versus vector group. (F) Representative images of xenografted tumors incorporating vector or pcDNA3.1‐RALYL transfection. Tumors are extracted from mice on Day 28 of tumor growth. Tumor volume is measured and calculated every 3 days since the first week post‐injection (*n* = 5, *p* < 0.01). (G) HE and IHC staining images of the tumors collected from the xenograft nude mice. Magnification: 200×. (H and I) Tumorigenesis in vivo in the RALYL group and the vector control group. (J) immunohistochemical staining of tumor tissue CRC, colorectal cancer; HE, hematoxylin and eosin; IHC, immunohistochemistry.

The full length of human RALYL cDNA was cloned into the pcDNA3.1 vector to overexpress RALYL in CRC cells. mRNA and protein expression were measured in SW480 and HCT116 transfected with overexpression or negative vector, and results were shown in Figure 2A,B that overexpression of RALYL was confirmed. Results of the cell viability assay indicated that overexpression of RALYL inhibited cell viability of both SW480 and HCT116 (Figure 2C). CRC cells transfected with the RALYL overexpression vector showed a high ability for monoclonal formation and cell proliferation based on clonal formation experiments and EdU staining (Figure 2D,E). As shown in Figure 2F, overexpression of RALYL suppressed CRC tumor growth in mice (Figure 2F). It was worth noting in the results of immunohistochemical staining that RALYL expression was negatively correlated with Ki67 expression in tumor tissue, which was consistent with that in human CRC tissue (Figure 2G). CRC cells transfected with RALYL overexpressing vectors formed smaller tumors in mice (Figure 2H,I).

### 3.3 MNK2 Alternative Splicing Plays a Significant Role in the Tumor Suppressive Function of RALYL

**FIGURE 3:**
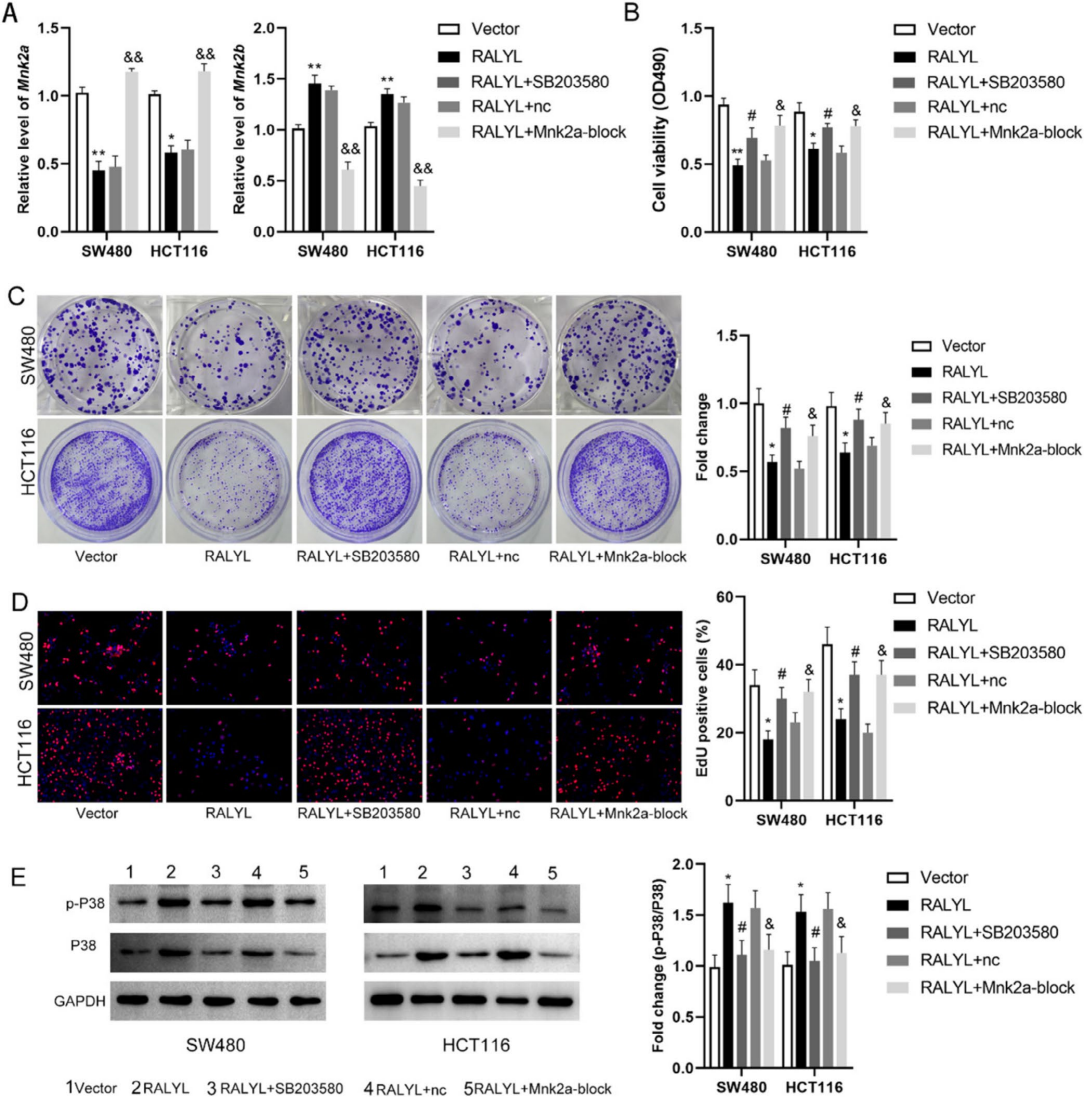
RALYL associated with MNK2 alternative splicing in CRC. (A) Relative expression of *MNK2a and MNK2b* in CRC‐treated cells using RT‐qPCR. (B) The proliferation of treated CRC cells using the MTT assay. (C) The colony formation of treated CRC cells using the colony formation assay. (D) Percentage of EdU‐positive cells in treated CRC cells. (E) Western blot analysis of p‐P38 and total P38 levels in the cell lysate of treated CRC cells. The quantification of p‐P38 protein levels is normalized to GAPDH. *n* = 3. *p < 0.05, **p < 0.01 versus vector group; #*p* < 0.05 versus RALYL group; &*p* < 0.05, &&*p* < 0.01 versus RALYL + nc group. CRC, colorectal cancer; nc, negative control of Mnk2a‐block.

To explore the relation of MNK2 alternative splicing with RALYL in CRC, the level of MNK2a and MNK2b was assayed in CRC cells transfected with RALYL overexpression or negative control vector with/without MNK2a block and treated with SB203580. The level of MNK2a was increased while MNK2b decreased in CRC cells transfected with the RALYL overexpression vector but was not affected by inhibition (Figure 3A). MNK2a block downregulated MNK2a while upregulated the MNK2b level in CRC cells transfected with the RALYL overexpression vector. Results of CCK8 demonstrated that the cell viability of RALYL overexpression cells was decreased, which was reversed by SB203580 and MNK2a block transfection (Figure 3B). It was similar to the results of cell viability that RALYL overexpression suppressed cell proliferation mediated by clone formation assay and EdU staining. Moreover, SB203580 and MNK2a block reversed the inhibitory effect of RALYL overexpression on the cell proliferation of CRC cells (Figure 3C,D). Results of further exploration showed that the relative expression of p‐P38 to P38 was upregulated in CRC cells transfected with the RALYL overexpression vector, which was inhibited by SB203580 and MNK2a block (Figure 3E).

### 3.4 RALYL Regulates MNK2 Alternative Splicing via HNRNPC in CRC

**FIGURE 4:**
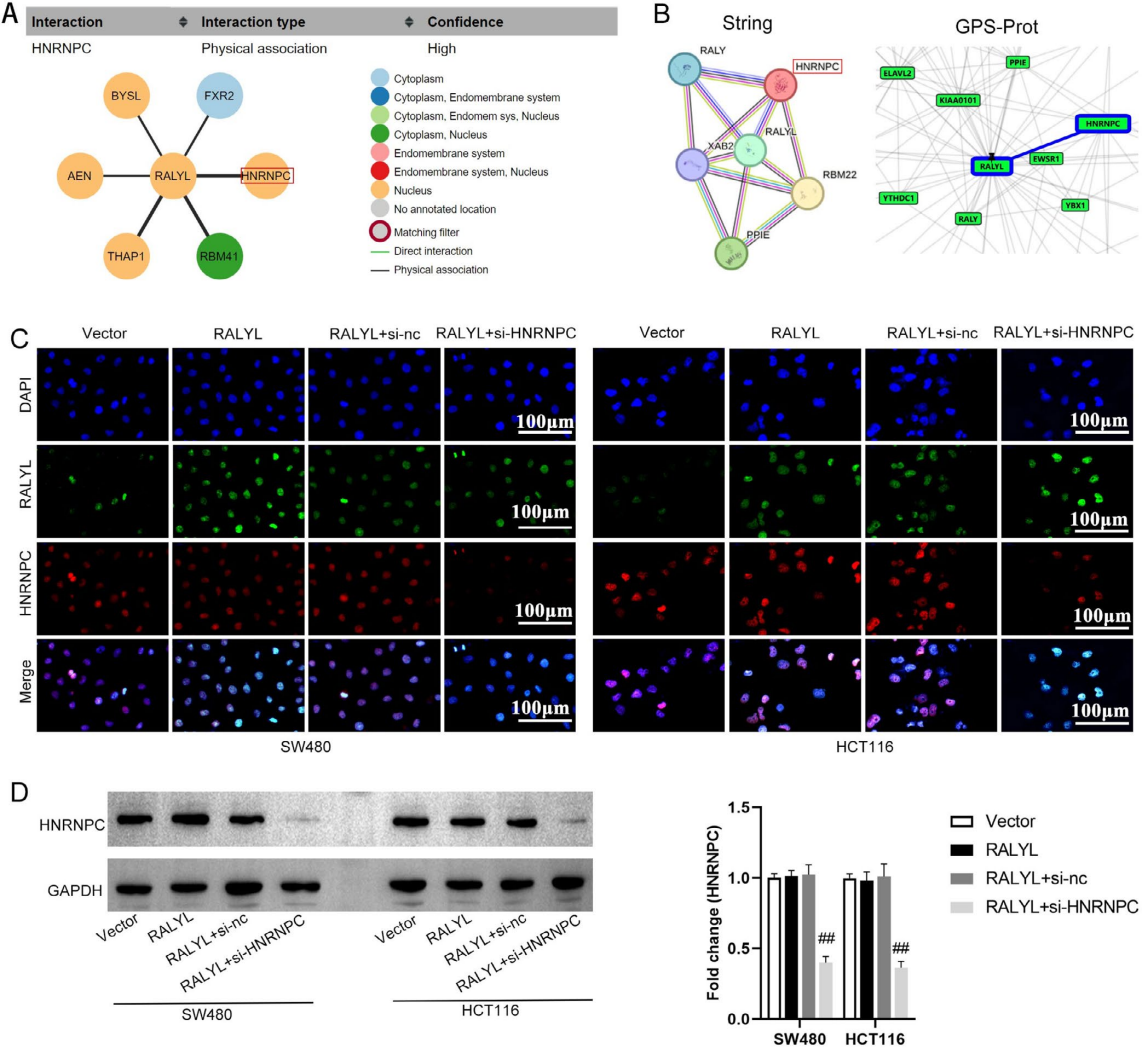
RALYL interaction with HNRNPC in CRC. (A) The interaction between RALYL and HNRNPC in The Interaction section of The Human Protein Atlas database. (B) The interacting protein HNRNPC of RALYL predicted by the String and GPS‐Prot databases. (C) The colocalization of HNRNPC of RALYL in CRC cells via co‐expression by immunofluorescence. (D) Protein level of HNRNPC in treated CRC cells using western blotting. *n* = 3, ##*p* < 0.01 versus RALYL + si‐nc group. CRC, colorectal cancer; nc, negative control; si, small interfering.

To investigate the mechanism of regulation of RALYL to MNK2 alternative splicing, bioinformatics analysis was conducted in the present study. It was shown in Figure 4A that there is a physical association of RALYL with HNRNPC, which is high in the nucleus. In addition, it is confirmed from String and GPS‐Prot analysis that RALYL interacts with HNRNPC (Figure 4B). Results of the immunofluorescence experiment showed that RALYL was coexpressed with HNRNPC in CRC cells (Figure 4C). Western blotting results indicated that the level of HNRNPC protein was not changed in RALYL overexpression cells, which were downregulated by si‐HNRNPC transfection (Figure 4D).

**FIGURE 5:**
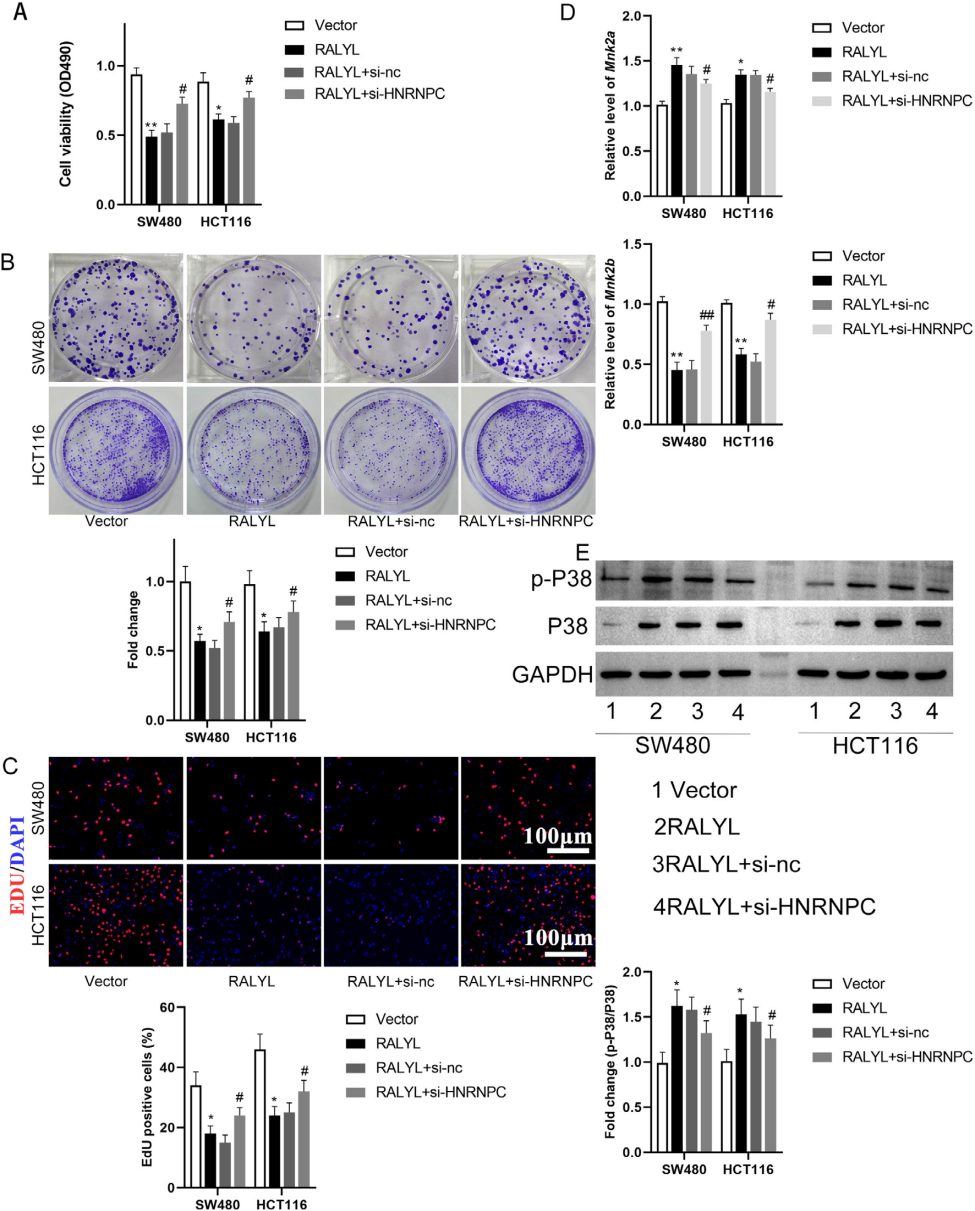
RALYL interaction with HNRNPC involved in P38 activation in CRC. (A) The proliferation of treated CRC cells using the MTT assay. (B) The colony formation of treated CRC cells using the colony formation assay. (C) Percentage of EdU‐positive cells in treated CRC cells. (D) Relative expression of *MNK2a and MNK2b* in CRC‐treated cells using RT‐qPCR. (E) Western blot analysis of p‐P38 and total P38 levels in treated CRC cells. *n* = 3. *p < 0.05, **p < 0.01 versus vector group; #*p* < 0.05, ##*p* < 0.01 versus RALYL + si‐nc group. CRC, colorectal cancer; nc, negative control; si, small interfering.

The role of RALYL associated with HNRNPC in CRC was explored in our study. Cell viability of RALYL overexpression CRC cells was promoted by si‐HNRNPC (Figure 5A). Cell proliferation of CRC cells from the clone formation assay and EdU staining was suppressed in RALYL overexpression cells, the inhibitory effect of which was reversed by si‐HNRNPC transfection (Figure 5B,C). It was worth noting that the levels of MNK2a were increased along with decreasing MNK2b levels in RALYL overexpression cells, and the effect of RALYL overexpression on both MNK2a and MNK2b was blocked by si‐HNRNPC transfection (Figure 5D). The effect of si‐HNRNPC on RALYL‐mediated P38 activation was explored in the following study results, which demonstrated that the relative expression of p‐P38 to P38 was upregulated in RALYL overexpression CRC cells, and the activated effect of RALYL on P38 activation was significantly inhibited by si‐HNRNPC (*p* < 0.05) (Figure 5E).

## 4 Discussion

The role of RALYL is variable in different kinds of cancer. Increasing expression of RALYL was observed and acted as an oncogene in hepatocellular carcinoma correlated with stemness [13, 14]. In another study, overexpression of RALYL suppresses the progression of ovarian clear cell carcinoma by inhibiting MAPK and CDH1 signaling pathways [5]. In the present study, we found that RALYL was hypermethylated. Overexpression of RALYL inhibited cell proliferation and tumor growth in CRC both in vitro and in vivo, suggesting that RALYL may function as a tumor suppressor gene in CRC.

To explore the molecular mechanism of RALYL in CRC, bioinformatics analysis indicated that RALYL may interact with HNRNPC, which has been shown in previous studies to promote the progression of CRC [15]. Moreover, HNRNPC is found to regulate cancer‐specific alternative cleavage and polyadenylation in CRC and is involved in tumor progression [16]. According to He et al., HNRNPC could regulate alternative splicing of MNK2 to facilitate gastric cancer peritoneal metastasis [10]. Results of our study indicated that MNK2 alternative splicing is essential for the tumor suppressive role of RALYL so that RALYL may inhibit CRC through regulating MNK2 alternative splicing by increasing MNK2a and decreasing MNK2b. In the present study, RALYL played a role in cancer inhibition through HNRNPC, which may be consistent with previous studies that HNRNPC promotes CRC progression involved in tumor growth [15]. However, overexpression of RALYL does not affect the protein level of HNRNPC, and co‐expression assay results showed that the binding between HNRNPC and RALYL was reduced. Therefore, RALYL may bind to HNRNPC to affect its structure to play a role in cancer inhibition.

As far as P38 pathway activation in cancer is concerned [17, 18, 19], in our study, RALYL, HNRNPC, and p38 were investigated, and the results indicated that HNRNPC and p38 are associated with the role of RALYL in CRC. Our findings suggest that RALYL may suppress CRC by activating p38. Mechanistically, RALYL may inhibit CRC by binding to HNRNPC to regulate MNK2 alternative splicing. Specifically, RALYL binds to HNRNPC to promote the splicing of MNK2 into MNK2a instead of MNK2b, which consequently activates the p38 MAPK signaling pathway and inhibits tumor proliferation in CRC.

## 5 Conclusion

Our study suggested that RALYL may inhibit CRC by binding to HNRNPC and promoting MNK2 splicing into MNK2a. This activation of the p38 MAPK signaling pathway leads to suppressed tumor proliferation, highlighting RALYL's potential as a therapeutic target for CRC.

## Author Contributions

**Feng Liu:** conceptualization (lead), data curation (lead), formal analysis (lead), funding acquisition (lead), investigation (lead), methodology (lead), project administration (lead), resources (lead), software (supporting), supervision (lead), validation (lead), visualization (supporting), writing – original draft (supporting), writing – review and editing (lead). **Zenghui Ma:** conceptualization (supporting), formal analysis (lead), investigation (supporting), methodology (lead), software (equal), validation (equal), writing – original draft (equal). **Jianbin Zhu:** conceptualization (supporting), investigation (equal), methodology (equal), supervision (supporting), writing – original draft (equal). **Min Chen:** investigation (supporting), project administration (supporting), resources (supporting), supervision (equal). **Guangming Wu:** investigation (supporting), project administration (supporting), resources (supporting), supervision (equal). **Xiaolong Liu:** formal analysis (supporting), investigation (supporting), methodology (supporting). **Zhanjun Hu:** data curation (supporting), resources (supporting). **Yufei Feng:** data curation (supporting), resources (supporting). **Xiaoliang Wang:** conceptualization (supporting), supervision (supporting), writing – original draft (supporting), writing – review and editing (supporting).

## Disclosure

The authors have nothing to report.

## Conflicts of Interest

The authors declare no conflicts of interest.

## Data Availability

RALYL promoter methylation levels in COAD and READ were obtained from UALCAN database at https://ualcan.path.uab.edu/. RALYL expression in COAD and READ was analyzed from GEPIA at http://gepia.cancer‐pku.cn/ and TCGA at https://portal.gdc.cancer.gov.
